# Supplementary material for: Are carbon emissions trading and green financial instruments synergistic? -Comprehensive quantitative research based on content analysis
Source: PLoS One. 2024 Mar 7;19(3):e0298601. doi: 10.1371/journal.pone.0298601 (PMC10919601; doi:10.1371/journal.pone.0298601)
Supplement: S1 Data — (ZIP) [file pone.0298601.s001.zip › Data/地级市Z_F工作报告.zip!/╕╩╦α/╠∞╦«╩╨/└┤╫╘╣1⁄2╓┌║┼í╛┬φ┐╦╩2╛▌═°í┐_2011.docx]

各位代表:

我代表市人民政府向大会报告工作。同时,根据《中共天水市委关于制定国民经济和社会发展第十二个五年规划的建议》,市政府书面提交了《天水市国民经济和社会发展第十二个五年规划纲要(草案)》,请予审议,并请各位政协委员及列席人员提出意见、建议。

“十一五”工作回顾

“十一五”时期,是我市经济社会发展极不平凡的时期,我们既经历了国际金融危机、“5·12”地震等重大考验,也迎来了国家建设关中—天水经济区、实施扩大内需政策等良好机遇。五年来,在省委、省政府和市委的坚强领导下,市政府坚持以科学发展观为指导,紧紧依靠全市人民,抢抓机遇,迎难而上,开拓创新,真抓实干,提前一年完成了“十一五”规划提出的主要目标任务,全市经济社会保持了快速健康发展的态势,进入了历史上发展最好最快的时期。

五年来,我们始终坚持把加快发展作为第一要务,大力实施“工业强市、农业稳市、商贸活市、旅游富市、科教兴市”五大战略,努力克服金融危机和重大自然灾害影响,经济运行质量明显提高,综合实力显著增强。

预计2010年实现生产总值298亿元,是“十五”末的 2倍,剔除物价因素,“十一五”年均增长11%。其中,第一产业增加值56亿元,年均增长9.4%;第二产业增加值115.8亿元,年均增长11.6%;第三产业增加值126.2亿元,年均增长11.1%。其他主要经济指标均保持了两位数增长。

创新天水经济技术开发区管理体制与机制,“一区多园”格局初步形成,园区科技孵化和产业聚集功能不断增强。坚持实施“10强50户”工程和振兴装备制造业“12341”行动计划,“工业强市”迈出新步伐。2010年全市规模以上工业实现增加值54.1亿元,“十一五”年均增长11.2%。主导产业不断发展壮大,装备制造业增加值由10.8亿元增加到19.9亿元,销售收入过亿元的企业由12户发展到24户,华天科技成功上市。加大工业投入,五年共组织实施工业项目402项,完成投资136.44亿元。企业技术进步与创新步伐明显加快,累计开发新产品300项,建成国家级企业技术中心4个,荣获中国驰名商标8个。国家高低压电器质量监督检验中心落户天水。县域工业经济实力明显增强,非公有制经济健康发展。循环经济起步良好,环境保护力度不断加大,节能减排目标全面实现。

在频繁遭遇自然灾害情况下,农业和农村经济持续快速发展。科技增粮取得实效,粮食产量稳步提高。果品、蔬菜、畜牧三大主导产业和中药材、花卉等特色产业规模不断壮大,新建城郊南山花牛苹果、威龙集团有机水果、渭河川道设施蔬菜、张家川和清水肉牛养殖示范园等一批特色产业基地,花牛苹果、秦安蜜桃、下曲葡萄、秦州大樱桃、清水核桃等果品品牌知名度进一步提升,果品、蔬菜种植面积和畜禽饲养量较“十五”末分别增长86.6%、82.5%和95.8%。设施农业、旱作农业等新技术大面积推广,航天育种基地建设加快。全市龙头企业发展到217家、农民专业合作社335家,农业产业化步伐明显加快。

认真贯彻落实扩大内需政策,家电下乡、以旧换新等活动深入开展,城乡消费市场繁荣活跃。2010年实现社会消费品零售总额125亿元,是“十五”末的2.6倍,年均增长20.9%。商贸服务业改造升级步伐加快,电子商务、连锁经营、物流配送、农超对接等新型业态快速发展。大力推进万村千乡、双百、新网和社区等市场工程建设,新建、改扩建市场110处,城乡市场体系日趋完善。深入实施“旅游产业三年翻番和质量提升计划”,麦积山、伏羲庙、南郭寺、玉泉观、大像山、水帘洞和清水温泉等重点景区景点的面貌得到显著改观。麦积山景区跻身国家地质公园,旅游产业体系更加完善。旅游接待人次年均增长20%,综合收入年均增长35.8%,相当于生产总值比重由“十五”末的5%上升到13.5%。

财政收支大幅度增长,2010年完成大口径财政收入47亿元,是“十五”末的2.9倍,年均增长23.8%。其中一般预算收入14.4亿元,年均增长23.49%;五县财政收入全部突破亿元大关。全市财政总支出120.6亿元,是“十五”末的4倍,年均增长32.11%,有力地保障了民生实事、重点项目建设和各项社会事业发展支出需要。坚持举办“金融活动周”,我市被评为“中国金融生态城市”。兰州银行、交通银行入驻天水,商业保险、邮政储蓄快速发展,金融对经济社会发展的支持力度不断加大。金融机构期末各项存贷款余额分别达到463.25亿元和214.99亿元,“十一五”年均增长22.6%和25.3%。

五年来,我们始终坚持发展抓项目不动摇,抢抓西部大开发、扩大内需、灾后恢复重建等机遇,全力以赴争项目、引资金,城乡基础设施建设取得新突破,发展基础更加坚实。

全市基础设施建设投入力度之大、开工项目之多,超过了历史任何时期。五年累计完成全社会固定资产投资746亿元,是“十五”期间的3.7倍,年均增长31.7%。共争取国家扩大内需项目397个,总投资51.91亿元,已全部开工建设,竣工率81.5%。1287个灾后恢复重建项目完成投资66.32亿元,城乡居民住房和教育、卫生、交通等灾后恢复重建任务基本完成。

组织实施交通建设项目2049个,天水军民合用机场开通民航,宝天高速公路建成通车,天定高速公路基本完工,宝天高速城区过境段、天平铁路等加快建设,一大批重大交通能源项目前期工作取得新突破。全市公路通车里程超过1万公里,实现了市县和县区之间三级以上公路连接,所有乡镇都通四级以上公路,所有行政村都通公路。通达、通畅和信息通讯基础设施建设突飞猛进,群众出行、信息交流更加便捷。

第三版《城市总体规划》批准实施,城市控制性规划覆盖率达到95%以上。深入开展“三城联创”活动,组织实施藉河和渭河风情线、伏羲城、瀛池大桥等市政基础设施重点项目72个,完成投资27亿元,城市综合服务功能显著增强。大力实施城市绿化工程,建成区绿化覆盖率达35.2%。历史名城、名镇、名村保护工作全面加强,一批古建筑、古民居、古遗址得到保护性维修。强化城市综合执法管理,市容市貌明显改观。五县县城和小城镇建设步伐不断加快,城镇化率达到33%。

新农村建设“百村示范、千村推进”工程和城乡一体化试点稳步推进,农村能源、安全饮水、农田基建、水利水保等工作全面加强,累计完成生态造林144.9万亩,兴修梯田74.58万亩,治理水土流失1155平方公里,新增节水灌溉面积17万亩,新建堤防101.3公里,建成户用沼气近10万户,解决了农村78.98万人饮水不安全问题。

五年来,我们始终坚持把改革开放作为推动发展的强大动力,积极推进重点领域和关键环节的改革,全方位扩大对外开放,发展环境不断优化,发展活力进一步增强。

农村土地流转和规模开发利用步伐加快,累计流转土地20.14万亩;集体林权制度改革试点工作全面完成,确权到户面积287.6万亩。以产权制度为核心的企业改革不断深化,市属企业改制面达到99.4%,粮食购销企业由86户精减到13户。部门预算、国库集中支付、政府采购、收入分配制度和国有资产管理等改革成效显著,县乡财政管理体制进一步完善。市、县区政府机构改革任务基本完成,事业单位改革有序推进。

《关中—天水经济区发展规划》全面实施,我市被列为经济区次核心城市,为跨越式发展提供了强有力的政策支持。按照“联结陇东南、融入大关中”的思路,积极主动加强与关中及陇东南地区的交流对接,初步建立了经济区长效合作机制,产业配套、经贸往来和文化旅游合作取得实质性进展。成功举办伏羲文化旅游节、轩辕文化旅游节、花牛苹果节、果品博览会等节会,积极开展陇右讲堂、纪念杜甫流寓陇右1250周年等文化交流活动,组团参加国内外重大经贸洽谈活动,全方位宣传推介天水,广泛开展招商引资。五年累计签约招商引资项目953项,到位资金167.2亿元。对外贸易在困境中突破,进出口总值由“十五”末的4189万美元增加到2.2亿美元。

五年来,我们始终坚持以人为本,高度重视和大力改善民生,加快发展社会事业,基本公共服务、社会保障及人民生活水平大幅提升。

积极推进素质教育,“普九”目标全面实现,青壮年文盲率下降到3.5%。以县为主的教育管理体制和义务教育经费保障机制初步建立,教育布局调整和基础教育课程改革深入推进。高中阶段教育普及程度大幅提高,中等职业教育快速发展,高等教育办学层次和规模不断提升。中小学校舍安全工程、特殊教育学校建设、儿童美德工程等加快实施,“十一五”累计教育总投入98亿元,是“十五”期间的2.72倍。大力推进科技进步与创新,我市被确定为国家先进制造高新技术产业化基地,连续五次荣膺“全国科技进步先进市”称号,科技创新工作连续6年名列全省第一,授权专利量突破千件大关,科技进步贡献率由“十五”末的42.8%提升到50.2%。创新人才工作机制,人才队伍不断发展壮大。文化建设步伐加快,《麦积圣歌》、《山里红》、《一画开天》、《邓宝珊将军》等彰显地域特色文化的文艺作品创作空前活跃,文物和非物质文化遗产保护工作全面加强,新建乡镇文化站74个、农家书屋1278个。文化旅游加快融合,产业规划开发格局初步形成。广播电视电影事业快速发展,3198个自然村实现通广播电视,城区开通手持电视,农村电影放映在全省率先实现数字化。全民健身活动广泛开展,竞技体育水平不断提升。医药卫生体制改革不断深化,疾病预防、妇幼保健等公共卫生全面加强,重点传染病得到有效控制,城乡医疗卫生服务体系进一步完善。新型农村合作医疗覆盖率达到95.47%,受益面不断扩大,城乡群众看病难、看病贵的问题得到初步缓解。人口和计划生育工作目标全面实现,人口自然增长率控制在7‰以内。第六次全国人口普查和第二轮修志工作进展顺利。人防、地震、消防、气象、档案、外事侨务等各项事业都取得了新成绩。

着力保障和改善民生,坚持每年办一批改善民生的好事实事,人民群众享受到了发展与改革带来的更多实惠。农村税费改革成果不断巩固,义务教育两免一补、粮食直补、农机具补贴、退耕还林补助、医疗救助、贫困生助学贷款等政策全面落实,妇幼、老龄和残疾人事业不断发展。认真落实全民创业、促进就业各项政策,累计新增城镇就业22万人,城镇登记失业率控制在4%以内。加强有组织的劳务输转,累计输转城乡富裕劳动力316.5万人次,创劳务收入163.4亿元。加大整村推进、连片开发力度,农村贫困面由22.14%下降到14.1%。城乡居民生活最低保障标准连续三年提高10%,农村养老保险试点工作有序推进,社会保障统筹能力全面提升,覆盖面进一步扩大。城乡低收入居民住房保障工作力度不断加大,共改造农村危旧房260万平方米,建设城镇廉租住房和经济适用住房186万平方米。城乡居民生活水平显著提升,城镇居民人均可支配收入达到11223元,农民人均纯收入达到2765元,年均分别增长10%和12.3%,实现了由基本解决温饱向全面建设小康社会迈进的历史性跨越。

五年来,我们始终坚持和谐社会建设,努力提升政府公共服务水平,不断强化社会管理和市场监管,社会大局稳定、人民安居乐业的良好局面得到进一步巩固和发展。

强化社会管理,各类自然灾害和突发公共事件应急管理体系逐步完善。有力、有序、有效地组织开展了“5·12”地震和洪涝灾害等抢险救灾工作,妥善安置受灾群众生产生活,积极承担并高效完成了中央和省上赋予我市支援陇南、舟曲等周边地区抢险救灾重大任务。深入开展“五五”普法和法律“六进”活动,依法治市进程全面推进。加强“平安天水”建设,依法严厉打击违法犯罪活动,社会治安持续好转。着力排查调处矛盾纠纷,积极开展市、县区主要领导信访接待日活动,充分利用市委书记、市长留言板等问政平台,一些社会热点难点问题得到及时有效解决。不断强化安全生产专项整治,安全生产形势总体平稳。加强食品、药品、知识产权等市场的专项整治和物价调控工作,市场秩序进一步规范。全面贯彻落实党的民族宗教政策,出台了《进一步加快张家川回族自治县经济社会发展的意见》,保持了民族团结、经济繁荣、宗教和顺的良好局面。

政府自身建设不断加强,重大决策咨询、听证、新闻发布等制度得到有效落实,电子政务加快推广,行政审批集中办理制度进一步完善。积极推进依法行政,坚持向人大及其常委会报告工作制度和规范性文件废改立备案制度,五年共办理人大代表建议938件、政协委员提案1369件,办结率均达到100%;废止规范性文件41件。认真执行廉政建设责任制,不断加大审计监督和行政监察力度,强化政风行风建设,公务员敬业勤政、责任效率、廉洁荣誉意识显著增强。

各位代表,刚刚过去的2010年,我们以保增长、扩内需、强基础、调结构、促和谐为主线,大力实施“1135”区域发展战略,经济社会平稳较快发展,确保了“十一五”目标的全面实现。与2009年相比,预计全市生产总值增长11%,大口径财政收入增长31.21%,全社会固定资产投资增长33.2%,规模以上工业增加值增长13.8%,社会消费品零售总额增长19%,城镇居民人均可支配收入增长13%,农民人均纯收入增长15%,城镇登记失业率、人口自然增长率、万元生产总值综合能耗均在控制指标以内。关中—天水经济区建设各项工作扎实推进,建立了陇东南四市招商引资项目推介合作机制,与关中各城市签订了20多项经贸、文化、旅游、科教、卫生等战略合作协议。重大项目建设取得新进展,争取国家和省上建设项目484项,总投资22.09亿元;组织实施市列重大建设项目50个,累计完成投资83.27亿元。交通、能源等重大项目加快建设,33个城市重点建设项目完成投资6.17亿元。天水经济技术开发区晋升为国家级经济技术开发区,社棠工业园总投资8.13亿元的基础设施建设全面启动,已入园企业13个,项目总投资47.26亿元。华天电子科技产业园、星火机床工业园开工建设,长城电工电器产业园和酒钢西部重工制管基地项目成功签约,西电高压电工触头生产基地、娃哈哈天水生产基地、中材水泥生产线二期等一批“引大引强”项目顺利实施,我市被评为“国家商标战略实施示范城市”。农业和农村工作亮点频出,天水农业高新技术示范区晋升为国家农业科技园区,“一城两园一集团”启动建设,秦州区被纳入全省“两江一水”生态保护与综合治理建设规划,特色优势产业基地建设和品牌培育全面加强。制定出台了加快发展旅游业的《实施意见》,旅游产业发展步伐进一步加快。麦积山景区晋升为国家5A级旅游景区,玉泉观、南郭寺、伏羲庙景区晋升为国家4A级旅游景区,全年接待游客人次和实现旅游综合收入均增长28.7%。承办和举办了公祭伏羲大典暨伏羲文化旅游节和第三届中国旅游电视艺术周,参加了西洽会、兰洽会、西博会、文博会等经贸文化活动,签约招商引资项目180项,总投资213亿元;当年实施招商引资项目143项,到位资金52.9亿元,同比增长29.6%。进一步加大民生实事的组织实施和督查工作力度,省上确定的14件、市上确定的12件改善民生实事全面落实。

各位代表,过去的五年,是我市历史上经济社会发展最快、城乡面貌变化最大、人民群众得到实惠最多的时期。“十一五”发展成就,既为“十二五”跨越发展奠定了坚实基础,也为我们做好今后的政府工作积累了宝贵经验,这就是:必须坚持把解放思想、转变观念作为加快发展的先导,勇于创新、勤于实践,创造性地做好各项工作;必须坚持以加快发展为第一要务,积极实施项目带动战略,努力推动经济社会跨越式发展;必须坚持统筹兼顾,促进城乡、经济社会、人与自然全面协调可持续发展;必须坚持以人为本,把提高人民生活水平作为根本出发点和落脚点,大力保障和改善民生;必须坚持深化改革、扩大开放,着力破解体制机制障碍,增强发展动力与活力;必须坚持强化社会管理,维护社会稳定,营造加快发展的环境;必须坚持依靠广大人民群众,凝聚各方面的智慧和力量,形成万众一心、狠抓落实的强大合力。

各位代表,五年取得的成绩来之不易,积累的经验弥足珍贵,创造的精神财富影响深远。这些成绩和经验的取得,凝聚着全市各族人民的汗水和智慧,得益于党中央、国务院的英明决策,得益于省委、省政府和市委的坚强领导,得益于历届市委、市政府打下的坚实工作基础,得益于市人大及其常委会的法律监督和工作监督,得益于市政协及各民主党派、工商联、无党派人士、人民团体、离退休老同志的关心帮助,得益于驻市各单位、驻市部队和社会各界的鼎力支持。在此,我代表市人民政府向全市各族人民,向所有关心、支持、参与天水改革发展的各界人士、天水籍在外乡亲,表示衷心的感谢并致以崇高的敬意!

各位代表,在看到成绩的同时,我们也清醒地认识到,我市经济社会发展中还面临一些不容忽视的矛盾和问题,主要是:经济总量小,人均水平低,外向度不高,实现跨越发展的压力与挑战巨大;经济结构性矛盾突出,特色优势产业规模水平低,新兴产业发育不足,新型工业化、农业产业化、信息化进程不快;城乡基础条件尚未得到根本性改善,资源、环境与人口压力日益增大;社会事业发展滞后,公共服务水平较低;城乡居民收入渠道单一,就业再就业形势严峻;社会管理中仍面临一系列新情况、新问题,维护稳定的任务十分艰巨;发展环境还不宽松,政府工作水平和效率还有待进一步提高。对于这些问题,我们将以百倍的努力,在加快发展、科学发展、和谐发展中逐步加以解决。
